# Establishment of a Visual Gene Chip Method for the Simultaneous Detection of Seven Waterfowl Virus Pathogens

**DOI:** 10.3390/v17030358

**Published:** 2025-02-28

**Authors:** Linjie Yan, Yafen Song, Tianshu Zhai, Qian Qiu, Jia Wang, Jinming Liu, Daiyue Lv, Xiaojie Huang, Huabin Cao, Chenghuai Yang, Yaqing Mao

**Affiliations:** 1 China Institute of Veterinary Drug Control, Beijing 100081, China; yanlaela0315@gmail.com (L.Y.); songyafen1@126.com (Y.S.); zhaitianshu1994@163.com (T.Z.); 13161248668@163.com (J.W.); liujinming@nwafu.edu.cn (J.L.); daiyuelv@gmail.com (D.L.); fjhxj168@126.com (X.H.); 2 Jiangxi Provincial Key Laboratory for Animal Health, Institute of Animal Population Health, College of Animal Science and Technology, Jiangxi Agricultural University, Nanchang 330045, China; 13647911394@163.com

**Keywords:** waterfowl, pathogen, molecular diagnostics, gene chip, visualisation, viruses

## Abstract

Goose parvovirus (GPV), duck enteritis virus (DEV), Muscovy duck parvovirus (MDPV), duck hepatitis A virus type 1 (DHAV-1), duck hepatitis A virus type 3 (DHAV-3), duck Tembusu virus (DTMUV), and novel duck reovirus (NDRV) are significant pathogens that spread extensively among waterfowl populations, causing economic losses for the waterfowl industry. In order to detect seven pathogens simultaneously, a visual gene chip for the detection of multiple waterfowl disease pathogens was developed in this study. The gene chip was capable of specifically amplifying GPV, DEV, MDPV, DHAV-1, –DHAV-3, DTMUV, and NDRV. The sensitivity results showed that the lowest detection limit of the gene chip was 1 copy/μL for single and mixed samples. The reproducibility and stability tests demonstrated that the gene chip developed in this experiment exhibited not only excellent reproducibility but also remarkable stability, remaining functional for a minimum of 180 days. Compared to qPCR methods, the results showed that the sensitivity of the gene chip was slightly better than that of the qPCR method in detecting both single and mixed pathogens of the seven viruses. In this study, a total of 210 clinical samples were detected by the gene chip and qPCR, respectively, and the results of the two methods had a concordance rate of 98.1~100%, with a kappa value of 0.952, indicating that the consistency of the two detection methods was good.

## 1. Introduction

Waterfowl farming holds significant economic and ecological importance on a global scale. Waterfowl products, including duck meat, goose meat, and duck eggs, serve as crucial sources of food in numerous countries, especially in China, the world’s largest producer of waterfowl, where ducks and geese are farmed on a world-leading scale [1]. However, with the rapid development of waterfowl aquaculture, outbreaks of infectious diseases have become increasingly serious, causing huge economic losses to the aquaculture industry and posing a potential threat to the health of wild birds and other animal groups.

GPV and MDPV are pathogens that affect waterfowl, particularly young goslings and Muscovy ducklings, leading to severe disease, especially in those under one week of age [2]. GPV mainly exerts its impact on geese, whereas MDPV principally affects Muscovy ducks. Despite this, both viruses are capable of inducing sickness in young ducks and geese. However, due to the antigenic disparities between them, the cross-protection afforded is restricted [3]. Duck enteritis virus (DEV), also known as duck plague virus (DPV), is a member of the Alpha-herpesvirus subfamily within the Herpesviridae family. Duck plague, caused by DEV, is a widespread infectious epidemic afflicting ducks, geese, and swans. It endows infected waterfowl with acute, febrile, and septic clinical symptoms. Characterised by high contagiousness and prevalence, duck plague can rapidly spread through waterfowl populations, posing a significant threat to the aquaculture industry [4]. Duck hepatitis A virus (DHAV) is the primary causative agent of duck viral hepatitis. Duck viral hepatitis is an acute and highly fatal infectious disease. The main clinical manifestations include mental depression, and before death, the ducks assume a horny-like posture. DHAV is a member of the small RNA virus family of avian hepatoviruses, and the virus consists of three different genotypes, namely genotype 1 (DHAV classic strain), genotype 2 (DHAV Taiwan new type), and genotype 3 (DHAV Korea new type). Infections in ducks in China are mainly caused by DHAV-1 and DHAV-3, and the prevalence of DHAV-2 has not yet been detected [5,6]. Duck Tembusu virus disease is an infectious disease caused by infection with duck Tembusu virus (DTMUV), which broke out in duck farms in coastal areas of China in 2010. Clinical manifestations of the disease include diarrhoea in chicks and neurological symptoms in adult ducks, haemorrhagic necrosis of the ovaries in laying ducks, a sharp decline in egg production, and death in severe cases, which has caused severe economic losses to China’s duck farming industry [7,8]. Novel duck reovirus (NDRV) is a double-stranded RNA (ds RNA) virus that is a member of the genus orthoreovirus in the family Orthoviridae. It can infect almost all types of waterfowl species, such as domestic ducks and geese. NDRV can induce splenic necrosis in ducks and geese. The pathological manifestations are mainly characterised by haemorrhagic necrosis of the liver and spleen. Currently, it is a prevalent disease on duck farms [9,10,11].

Group or endemic waterfowl epidemics have become particularly common in China. These epidemics mostly occur in the form of mixed infections, which makes treatment more difficult and leads to more severe morbidity conditions, as well as higher morbidity and mortality rates. At present, in response to the above pathogens, scholars at home and abroad have established common PCR [12,13], Quantitative Real-time polymerase chain reaction (qPCR) [14,15], and other detection methods [16]. However, there is no detection method that can simultaneously detect seven waterfowl viruses. There is, therefore, an urgent need to develop a rapid and sensitive method for the detection of waterfowl pathogens.

A gene chip is a type of biochip, also known as a DNA chip, DNA microarray, or oligonucleotide array [17]. Gene chip detection technology is the current most advanced means in the field of clinical disease gene function research, and will also be an important development direction in the field of disease diagnosis and life science research in the future [18,19]. Advances in microarray technology have made large-scale parallel mining of biological data possible, and biochips offer a wide range of uses for hybridisation-based expression monitoring, polymorphism detection, and genome-scale genotyping [20,21]. In recent years, the combination of visual colorimetric technology and traditional biochips has greatly broken through the limitations of traditional gene chips in clinical applications. Through the unique chromogenic reaction, colour spots can be directly observed by the naked eye, providing an intuitive and convenient means of observation for experimenters [22]. Compared with traditional detection methods, the visual gene chip has the advantages of high throughput, good specificity, high sensitivity, and simultaneous detection of multiple viruses. In addition, it does not require other complex or expensive equipment. After the reaction is completed, the reaction at the probe site can be observed directly with the naked eye to determine whether the sample contains the disease-causing nucleic acid to be detected, which greatly improves the detection efficiency and greatly facilitates the rapid monitoring and control of diseases.

In this study, we developed a visual gene chip that can be used to detect multiple waterfowl disease pathogens. This detection method has the characteristics of convenience, high efficiency, and high sensitivity in detecting multiple pathogens of waterfowl. It is a new advanced tool for the rapid diagnosis, prevention, and control of waterfowl diseases.

## 2. Materials and Methods

### 2.1. Virus Strains

Goose parvovirus, duck enteritis virus, duck hepatitis A virus type 1, duck hepatitis A virus type 3, duck Tembusu virus, novel duck reovirus, Dastv, and DadV-3 were provided by the China Institute of Veterinary Drug Control.

### 2.2. Primers and Probes

To ensure the effectiveness of the primers and probes used in the gene chip assay method, all available sequences of GPV, DEV, MDPV, DHAV-1, DHAV-3, DTMUV, and NDRV were obtained from the GenBank database and analysed. Prime5 (5.0) software was used to design the primers and probes for GPV, DEV, MDPV, DHAV-1, DHAV-3, DTMUV, and NDRV. The design was based on the *NS* gene of GPV, the *UL6* gene of DEV, the *VP* gene of MDPV, the *VP1* gene of DHAV-1, the *VP1* gene of DHAV-3, the *E* gene of DTMUV, and the *S4* gene of NDRV (Table 1).

### 2.3. Construction of Plasmid Standards

Nucleic acids were extracted from seven viral samples according to the instructions of the viral genome RNA/DNA extraction kit (Takara Biomedical Technology Co., Ltd, Beijing, China). The 119–197 bp fragments containing the amplified fragments from each pathogenic primer in Table 1 were used as target fragments inserted into the pMD19-T vector (Takara Biomedical Technology Co., Ltd, Beijing, China) and confirmed by DNA sequencing. The plasmid copy number was calculated, and the plasmids were diluted to 1 × 10^8^ copies/μL.

### 2.4. Multiplex PCR Amplification Design and Optimisation

Using the extracted nucleic acids as templates, amplification reactions were carried out with the HiScript II One Step RT-PCR Kit (Vazyme Biotech Co., Ltd, Nanjing, China). Seven pairs of primers were placed in the same PCR amplification system. The annealing temperature, extension time, and primer dosage of the reaction were optimised to determine the best conditions for amplification of the target gene. The reaction system is shown in Table 2. The reaction conditions were as follows: 50 °C for 30 min, 94 °C for 4 min, 94 °C for 30 s, 56 °C for 30 s, 72 °C for 20 s, 5 cycles, 94 °C for 30 s, 54 °C for 30 s, 72 °C for 20 s, 35 cycles, 72 °C for 10 min, 95 °C for 5 min.

#### 2.4.1. Optimisation of the Annealing Temperature for the Sevenfold PCR Amplification Reaction

Since the optimal annealing temperatures of the PCR primers vary for each pathogen, it is necessary to optimise the annealing temperature for the PCR amplification in order to screen out the optimal annealing temperature that can satisfy the specificity of the multiplex PCR amplification while possessing high efficiency. Using the mixed plasmids of the seven viruses as templates, the annealing temperature for the PCR amplification was optimised according to the amplification system described above. In addition, considering that the PCR amplification requires the simultaneous amplification of the target fragments of the seven pathogens, the use of a two-step cycle may give better amplification results. The annealing temperatures were set at 54 °C, 56 °C, 68 °C, 60 °C, 54/56 °C, 54/58 °C, 56/58 °C, and 56/60 °C. After the amplification products were subjected to 1% agarose gel electrophoresis, the electrophoretic bands at each annealing temperature were compared and analysed to determine the optimal annealing temperature for the PCR amplification.

#### 2.4.2. Optimisation of the Extension Times for the Sevenfold PCR Amplification Reactions

The extension time of the PCR amplification was experimentally optimised using the sevenfold hybrid plasmid as a template. Four different extension times of 10 s, 15 s, 20 s, and 30 s were set for PCR amplification. After 1% agarose gel electrophoresis of the amplified products, the electrophoretic bands were analysed and compared at each time setting to determine the optimal extension time for the PCR amplification.

#### 2.4.3. Optimisation of Primer Concentrations for the Sevenfold PCR Amplification Reactions

To optimise the numbers of upstream and downstream primers in the sevenfold PCR amplification system, each primer of the sevenfold PCR amplification was selected with an initial concentration of 100 μmol/L for optimisation. Using the sevenfold mixed plasmid as a template, different final primer concentrations of 0.05 μmol/L, 0.1 μmol/L, 0.2 μmol/L, and 0.4 μmol/L were set for PCR amplification. The amplified products were subjected to 1% agarose gel electrophoresis, and the electrophoretic bands corresponding to each primer concentration were compared and analysed to determine the optimal primer concentration for the PCR amplification.

### 2.5. Preparation of Gene Chip

**Figure 1:**
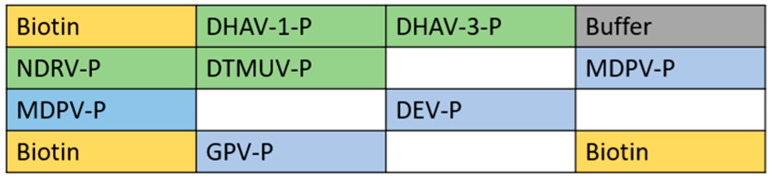
Arrangement of chips. Biotin is the positive QC for the gene chip; buffer is the negative QC for the gene chip.

The probes were diluted with phosphoric acid buffer to a final concentration of 6 μmol/L. The dispenser parameter was set to 100 drops, and the probes were dispensed onto the nanomembrane in order of chip arrangement. After sampling, the membrane substrate was dried overnight at 40% to 60% humidity and 22 to 25 °C and stored at 2 to 8 °C. The chip arrangement is shown in Figure 1.

### 2.6. Determination of Test Results

Negative QC points appear colourless; in contrast, positive QC points display a blue-purple colour that is significantly more intense than that of the negative QC points, and at least one of the three positive QC points shows colour as a valid test result. If the probe point where the tested sample is located shows no colour, it is negative; conversely, if the probe point corresponding to the tested sample displays a blue-purple colour, it is positive.

### 2.7. Gene Chip Hybridisation Condition Optimisation

#### 2.7.1. Gene Chip Hybridisation Time Optimisation

Hybridisation reaction time is an important factor affecting the accuracy of microarray detection results. By setting the hybridisation time to 30 min, 20 min, 15 min, 10 min, and 5 min, respectively, the signal strength, clarity of the reaction spots, and cleanliness of the background were analysed by observing the detection results under different hybridisation time conditions. The results were analysed and compared to determine the optimal hybridisation time for microarray detection.

#### 2.7.2. Optimisation of SA-HRP Dilution Concentration

The target amplified fragments were obtained by PCR amplificationas described in 2.4, and the PCR products were hybridised to the gene chip. To optimise detection, different concentrations of SA-HRP working solution were used for subsequent reaction processing. The SA-HRP working solution was diluted to 1:2000, 1:4000, 1:6000, 1:10,000, and 1:16,000 with Solution A. After reacting each concentration of SA-HRP working solution with the chip, the final dilution concentration of SA-HRP working solution was determined by observing the clarity of the reaction spots and the cleanliness of the background in the hybridisation results, and the results were analysed and compared.

### 2.8. Specificity Testing of the Gene Chip

Recombinant positive plasmids of GPV, DEV, MDPV, DHAV-1, DHAV-3, DTMUV, and NDRV, along with viral nucleic acids of Dastv, DadV-3, and a negative control (serum from healthy ducks), were used as templates for PCR amplification. Subsequently, the PCR amplification products were hybridised to the gene chip in a colorimetric reaction to validate the specificity of the gene chip.

### 2.9. Sensitivity of Single Positive Plasmids in Gene Chip

The concentration of each positive plasmid was determined using an ultra-micro UV spectrophotometer. Each positive plasmid was diluted to 1.0 × 10^8^ copies/μL, a 10-fold multiplicative dilution was used for PCR amplification, and the sensitivity of the chip for a single positive plasmid was determined after hybridisation of the PCR amplification products with the chip for colour development reaction.

### 2.10. Sensitivity of Gene Chip to Mixed-Positive Plasmids

Equal volumes of each positive plasmid, which had been diluted to 1.0 × 10^8^ copies/μL, were mixed, a 10-fold multiplicative dilution of the mixed plasmid was performed for PCR amplification, and the sensitivity of the chip to the mixed-positive plasmid was determined after hybridisation of the PCR amplification product to the colour development reaction chip.

### 2.11. Gene Chip Reproducibility Testing

To verify the reproducibility of the microarray, microarray experiments were performed by different experimenters at different time points using the amplification products of the same quality control sample (positive recombinant plasmid). In each experiment, the PCR amplification conditions were the same as those used for chip hybridisation and colour development to ensure consistency of conditions. The experiments were divided into intra-batch reproducibility testing within the same batch and inter-batch reproducibility testing between different batches. The intra-batch and inter-batch reproducibility of the microarray was verified by analysing and comparing the results.

### 2.12. Gene Chip Stability Testing

To test the stability of the gene chip assay, the assay was performed using mixed-positive samples of seven viruses on days 0, 30, 60, 90, 120, and 180 after chip manufacture, and the stability of the chip under different storage times was verified by analysing and comparing the results.

### 2.13. Clinical Sample Detection

During epidemiologic surveillance, we systematically collected clinical samples, including throat swabs, anal swabs, and organ samples (livers, lungs, and hearts) from poultry farms in Shandong, Jiangxi, and Fujian Provinces in China. These biological materials were then subjected to virus isolation in our biosafety level 2 (BSL-2) facility following strict biosafety protocols. The PCR products were tested by the gene chip and qPCR methods, respectively, to calculate the compliance rate.

## 3. Results

### 3.1. Recombinant Positive Plasmid Construction

**Figure 2:**
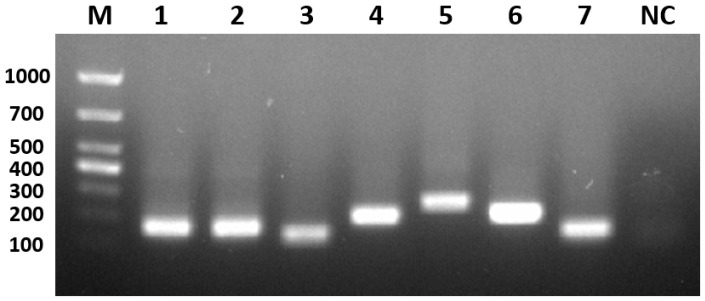
PCR products of recombinant positive plasmids by agarose gel electrophoresis. M: marker; 1: GPVXP; 2: DEVXP; 3: MDPVXP; 4: DHAV-3XP; 5: DTMUVXP; 6: DHAV-1XP; 7: NDRVXP; NC: negative control.

The nucleic acid of each viral fluid was extracted as a template, and PCR amplification was performed to obtain amplified bands of the same size as the target fragment. The purified amplified product, which was purified by gel recovery, was ligated into the vector, then transformed and identified by bacteriophage PCR. The plasmid was extracted from the PCR-positive samples and sent to a sequencing company (Taihe Biotechnology Co., Ltd, Beijing, China) for sequencing. The sequencing results were as expected and confirmed the successful construction of seven recombinant positive plasmids, named GPVXP, DEVXP, MDPVXP, DHAV-1XP, DHAV-3XP, DTMUVXP, and NDRVXP (Figure 2). The concentration of each positive plasmid was determined with an ultraviolet spectrophotometer, and each positive plasmid was diluted to 1.0 × 10^8^ copies/μL as the positive plasmid standard.

### 3.2. Optimisation of PCR Amplification Annealing Temperature

**Figure 3:**
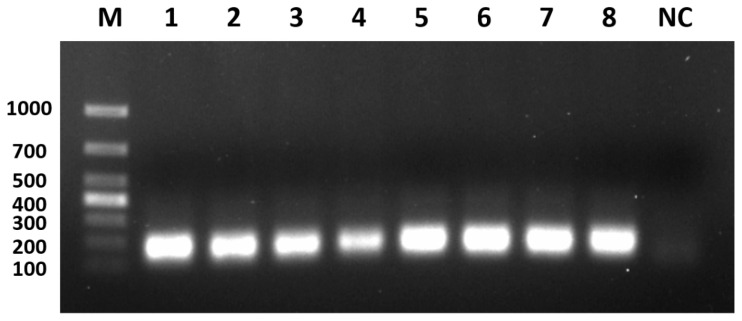
Optimisation of the annealing temperature for the PCR amplification. M: marker; 1: 54 °C; 2: 56 °C; 3: 58 °C; 4: 60 °C; 5: 54/56 °C; 6: 54/58 °C; 7: 56/58 °C; 8: 56/60 °C; NC: negative control.

The optimisation results are shown in Figure 3. The best amplification results were obtained at an annealing temperature of 54 °C/56 °C, with a clean background and clear destination bands that were significantly brighter than the background compared to other destination bands, specifically with 5 reaction cycles at 56 °C followed by 35 reaction cycles at 54 °C. Therefore, the 54 °C/56 °C annealing temperature was selected as the optimal annealing temperature for the reaction system.

### 3.3. Optimisation of PCR Amplification Extension Times

**Figure 4:**
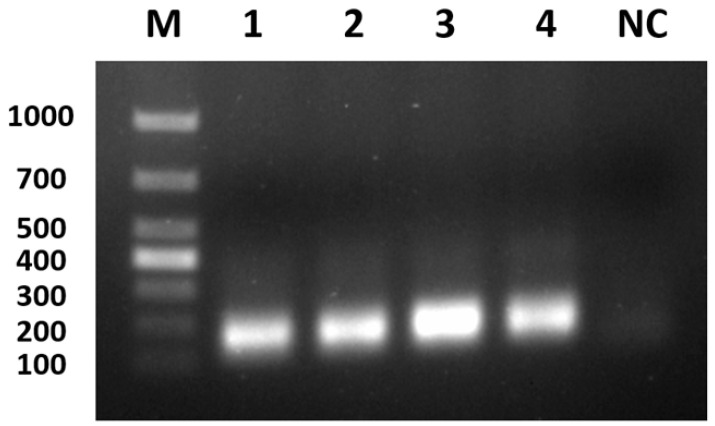
Optimisation of PCR amplification extension times. M: marker; 1: 10 s; 2: 15 s; 3: 20 s; 4: 30 s; NC: negative control.

The optimisation results are shown in Figure 4. When the post-delay time was 20 s, the amplification effect was the best, the background cleanliness was high, and the destination bands were clear. Moreover, the brightness of other destination bands was significant compared with the background. Therefore, an extension time of 20 s was chosen as the optimal extension time of the reaction system.

### 3.4. Optimisation of Primer Concentrations for PCR Amplification

**Figure 5:**
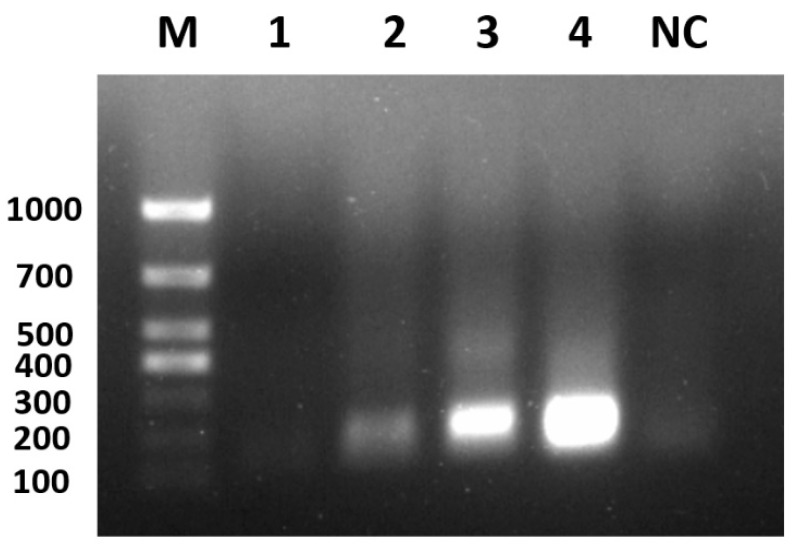
Optimisation of primer concentrations for PCR amplification. M: marker; 1: 0.05 μmol/L; 2: 0.1 μmol/L; 3: 0.2 μmol/L; 4: 0.4 μmol/L; NC: negative control.

The results of the optimisation are shown in Figure 5. The target band was clearest and the best amplification effect was achieved when the primer concentration was 0.4 μmol/L. Therefore, 0.4 μmol/L primer concentration (i.e., the amount of each primer was 0.1 μL/reaction) was selected as the optimal primer concentration of the reaction system.

### 3.5. Optimisation of Hybridisation Times for Gene Chips

**Figure 6:**
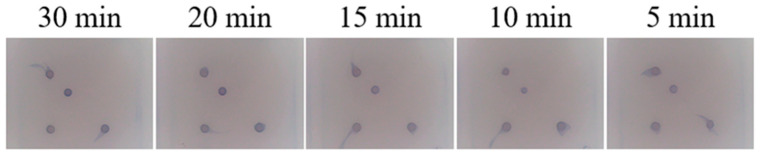
Optimisation of hybridisation times for gene chips.

The results of the optimisation are shown in Figure 6. By observing the detection results under different hybridisation time conditions and analysing and comparing the signal strength, clarity, and cleanliness of the background of the response spot, it can be seen that the signal strength of the response spot is strongest when the hybridisation time is 20 min, and the clarity and cleanliness of the background are better than in other results. Therefore, a hybridisation time of 20 min was selected as the optimal hybridisation time for the chip reaction system.

### 3.6. Optimisation of SA-HRP Concentration for Gene Chips

**Figure 7:**
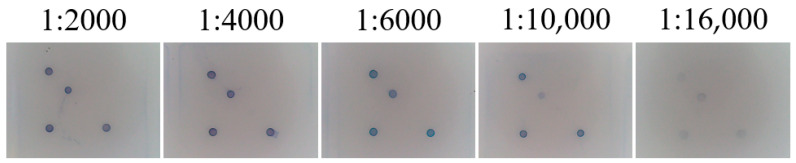
Optimisation of SA-HRP concentration for gene chips.

The results of the optimisation are shown in Figure 7. Observing the detection results under different SA-HRP concentration conditions, the hybridisation signals of the chip at 1:2000 dilution appeared blurred and the background cleanliness was low, the hybridisation signals of the chip at 1:4000 and 1:6000 dilution were strong and clear with the best background cleanliness, and the hybridisation signals of the chip at 1:10,000 and 1:16,000 dilution were obviously attenuated.

### 3.7. Specificity of the Gene Chip

**Figure 8:**
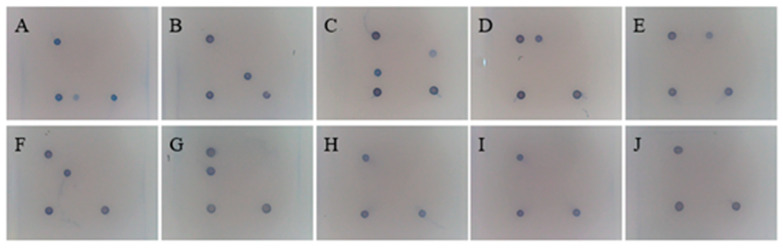
Specificity of the gene chip. (**A**): GPV; (**B**): DEV; (**C**): MDPV; (**D**): DHAV-1; (**E**): DHAV-3; (**F**): DTMUV; (**G**): NDRV; (**H**): Dastv; (**I**): DadV-3; (**J**): negative control.

The results are shown in Figure 8. Negative QC sites did not react, positive QC sites did react, and the test results were established. All seven pathogen probe sites showed specific binding without cross-reactivity, indicating that the specificity of each pathogen probe used in the chip is good.

### 3.8. Sensitivity of Single Plasmid Standards for Gene Chips

**Figure 9:**
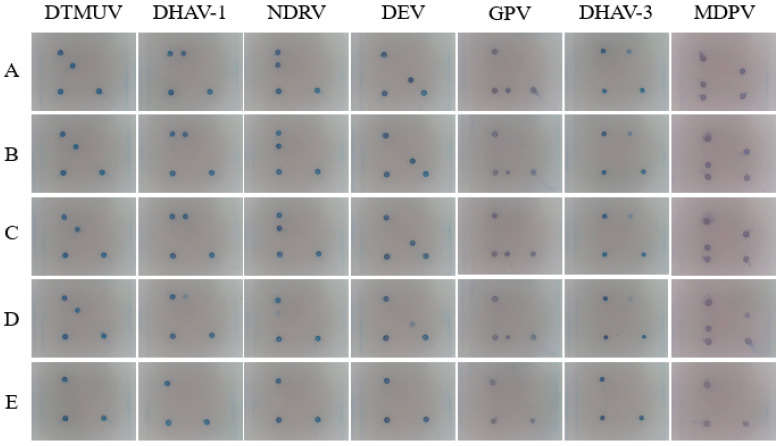
Sensitivity of single plasmid standards for gene chips. (**A**): 1.0 × 10^3^ copies/μL; (**B**): 1.0 × 10^2^ copies/μL; (**C**): 1.0 × 10^1^ copies/μL; (**D**): 1.0 × 10^0^ copies/μL; (**E**): negative control.

The results are shown in Figure 9. At the negative QC points, no response was detected, while at the positive QC points, a response was observed. This confirmed the validity of the test results. The single sample sensitivity of GPV, DEV, MDPV, DHAV-1, DHAV-3, DTMUV, and NDRV was 1.0 × 10^0^ copies/μL.

### 3.9. Sensitivity of Mixed Plasmid Standards for Gene Chips

**Figure 10:**
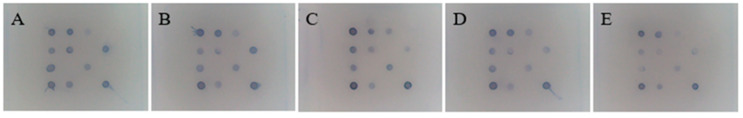
Sensitivity of mixed plasmid standards for gene chips. (**A**): 1.0 × 10^4^ copies/μL; (**B**): 1.0 × 10^3^ copies/μL; (**C**): 1.0 × 10^2^ copies/μL; (**D**): 1.0 × 10^1^ copies/μL; (**E**): 1.0 × 10^0^ copies/μL.

The results are shown in Figure 10. At the negative quality control (QC) points, no reaction occurred, whereas at the positive QC points, reactions were observed, thus validating the test results. The seven plasmids were mixed and diluted to 1.0 × 100 copies/μL at 10-fold multiplicity; the sensitivity of the seven plasmid-mixed samples of GPV, DEV, MDPV, DHAV-1, DHAV-3, DTMUV, and NDRV was 1.0 × 10^0^ copies/μL.

### 3.10. Reproducibility of the Gene Chip

To verify the reproducibility of the gene chip assay, microarray experiments were performed by different operators at different time points using different dilutions of amplification products from the same quality control sample (positive recombinant plasmid). Reproducibility was verified within batch number CH240828001 and within batch numbers CH240620001 and CH240718001. In each experiment, the PCR amplification conditions were the same as those used for chip hybridisation and colour development to ensure consistency of conditions. The results are shown in Table 3. The consistency of the assay results within and between batches reached 100%, indicating that the method has good reproducibility.

### 3.11. Stability of the Gene Chip

**Figure 11:**
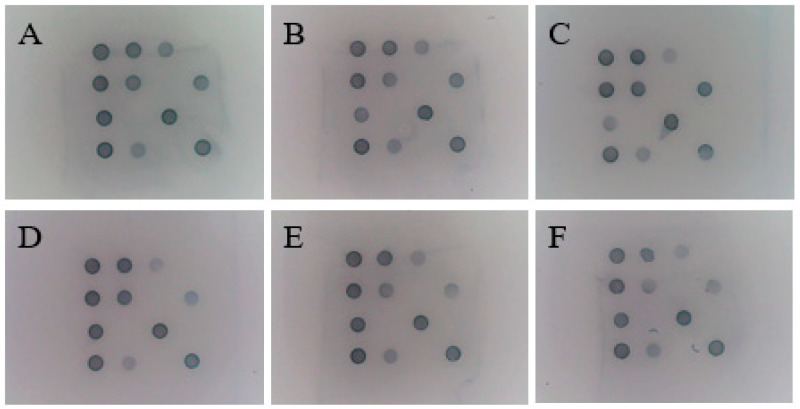
Stability of the gene chip. (**A**): 0 days; (**B**): 30 days; (**C**): 60 days; (**D**): 90 days; (**E**): 120 days; (**F**): 180 days.

Using the sevenfold mixed-positive plasmids as templates, stability tests were performed at 0, 30, 60, 90, 120, and 180 days after the completion of chip production. The results are shown in Figure 11. Under the storage conditions of 2 °C~8 °C, the gene chip can be stably stored for at least 180 days. This demonstrates that the method has good long-term storage stability.

### 3.12. Comparison of Gene Chip Detection Methods with Real-Time PCR Methods

A total of 210 clinical samples suspected of being positive for the pathogen were used in this study via gene chip and qPCR detection methods, respectively. The results are shown in Table 4, with a concordance rate of 98.1% to 100% and a kappa value of 0.952. These data indicate a good agreement between the two methods.

## 4. Discussion

GPV, DEV, MDPV, DHAV-1, DHAV-3, DTMUV, and NDRV are common pathogens that pose a great threat to the waterfowl farming industry. In addition, mixed infections with the seven pathogens mentioned above may occur in some cases, which increases the complexity of detection [23,24,25]. For the above waterfowl disease pathogens, commonly used detection methods such as the RT-PCR and qPCR methods have the problems of easy contamination and causing false positives. In addition, qPCR methods can usually detect only 1–4 pathogens, which means a low detection efficiency, and this limitation is especially prominent in the prevention and control of frequent waterfowl epidemics. Therefore, it is particularly important to develop techniques that can simultaneously detect mixed infections of multiple pathogens. In recent years, with the continuous development of detection technology, gene chip technology has been widely used in drug development, gene mutation, and gene expression [26,27,28,29,30]. This technique uses the principle of complementary base pairing to allow hybridisation of treated samples with spotted nucleic acid probes. Meanwhile, in this study, the Biotin-SA-HRP/TMB chromogenic system was used to add biotin, which has high affinity with SA-HRP, to the primers, and the nanomembrane was used as the probe carrier, so that when the target fragment bound to the probe, a blue reaction dot appeared and the detection result could be identified with the naked eye, and the strength of the hybridisation signals could be analysed to determine whether the samples contained the target gene fragments and their contents. The strength of the hybridisation signal can be analysed to determine whether the sample contains the target gene fragment and its content. In this study, a novel gene chip was developed to simultaneously detect the pathogens of these seven waterfowl infectious diseases and to recognise the results with the naked eye. This development provides strong support for on-site diagnosis of the diseases.

The process of gene chip detection technology includes the preparation of the chip, the preparation of the sample to be tested, and the signal detection and analysis of the hybridisation reaction between the sample and the chip. The biological reaction between the test sample and the chip is a key link in the signal detection and subsequent analysis of the gene chip. Among the factors involved, the size of the gene probe on the chip and the length of the target gene fragment have an important influence on the intensity of the hybridisation reaction signal of the chip [31]. In addition, factors such as PCR reaction conditions, chip hybridisation time, and SA-HRP concentration affect the strength of the positive chromogenic signal in actual experimental operations [32]. In this experiment, through the comparative analysis of the sequences of each pathogen strain in GenBank, the probes and primers were designed by selecting the conserved regions of their specificity, and several pairs of specific primers and probes were rapidly designed by using Premier 5 software to select the primers and probes with suitable primer length and GC content, and seven pairs of suitable specific primers and probes were screened to improve the accuracy and specificity of the assay. The probes designed in this experiment were all less than 30 bp in length, and the amplification products were all less than 200 bp. The optimised PCR amplification conditions were as follows: annealing temperature of 54 °C/56 °C, extension time of 20 s, primer concentration of 0.4 μmol/L, optimised microarray hybridisation time of 20 s, and SA-HRP concentration of 1:4000 to 1:6000. This improved the amplification efficiency, saved on experimental time and cost, and also ensured the accuracy of the test results and the clarity of the reaction signals. In addition, the gene microarray method developed in this study can simultaneously detect seven waterfowl pathogens in only 2.5 h, and the stability of the microarray can be maintained at low temperatures for at least 6 months, according to the results of this study. The microarray method established in this experiment is highly sensitive, and the detection limit of the seven common waterfowl viruses reached 1 copy/μL, which is higher than those of some existing qPCR detection methods. From the compliance rate of the gene chip and qPCR methods, the compliance rate for all pathogen detection reached 98.1% and the kappa value was 0.925, which indicated more positive samples in the detection of clinical samples, reflecting the advantage of the high sensitivity of the gene chip method and indicating that the visual gene chip can be used for the detection of clinical samples. Compared with traditional detection methods, the gene microarray has the advantages of easy operation, short time, intuitive result determination, and suitability for the simultaneous detection of multiple pathogens, which is especially useful in application scenarios such as those with a huge sample volume, a wide range of species, and the need to detect a large number of different pathogens. The research on and establishment of this assay are intended to provide a new and advanced means of rapid diagnosis, prevention, and control of the above diseases in waterfowl production.

## Figures and Tables

**Table 1:** Primers and probes used in this study.

Virus	Primer and Probe	Sequence (5′→3′)	Product Size (bp)
GPV	Forward	AATTGTTCYCATCAGTYGCTC	153
	Reverse	ARTTTGCYTTCTCACATTCCATAC	
	Probe	CCTGTGACTCCTCAGAACTCCCCT	
DEV	Forward	GGCCAGGGAGTTTATAATTCGG	161
	Reverse	GCTATATGTCGTGCATCTAACCC	
	Probe	CTGCCATACGWCAAATCCAGGCGAC	
MDPV	Forward	AAGCTACAACAACCACATSTAC	119
	Reverse	GGCAGTGRAATCTGTTGAAAT	
	Probe	ATCACAAGCGGAACAAACCCAGAC	
DHAV-1	Forward	TATGGGYCTCAAAAAGCCAGC	192
	Reverse	CTAAGGCAAAATCTGAAGTRCCC	
	Probe	TTCCACTCCCTGCTCCCACYTCC	
DHAV-3	Forward	TATTCTGTTACRCCBTTACGCCC	156
	Reverse	TGRTGCAGGCARTGGRAA	
	Probe	CGACCCATGCCAGYRTYTCAGGG	
DTMUV	Forward	ACTGGTTTCATGAYCTCAACTTACC	197
	Reverse	CATTTCCARTTTGYTTCCAGAGT	
	Probe	ACRGGGTCATCAGCGGGGACG	
NDRV	Forward	CGCCTGATACTTTCCCTCCT	124
	Reverse	GGCGTCTCAACACCACCAC	
	Probe	TCAAATCCCTCCAAAGCG	

**Table 2:** Sevenfold sample amplification system.

Sevenfold System Components	μL/Portion
ddH_2_O	5.85
GPV-F (100 μM)	0.1
GPV-R (100 μM)	0.1
DEV-F (100 μM)	0.1
DEV-R (100 μM)	0.1
MDPV-F (100 μM)	0.1
MDPV-R (100 μM)	0.1
DHAV-1-F (100 μM)	0.1
DHAV-1-R (100 μM)	0.1
DHAV-3-F (100 μM)	0.1
DHAV-3-R (100 μM)	0.1
DTMUV-F (100 μM)	0.1
DTMUV-R (100 μM)	0.1
NDRV-F (100 μM)	0.1
NDRV-R (100 μM)	0.1
2 × One Step Mix	12.5
One Step Enzyme Mix	1.23
Template	4
Total volume	25

**Table 3:** Gene chip intra- and inter-lot reproducibility.

Batch Code	1.0 × 10^3^	1.0 × 10^2^	1.0 × 10^1^	Negative Control	Total	Compliance Rate
CH240620001	9	9	9	9	36	100%
CH240718001	9	9	9	9	36	
CH240828001-1	3	3	3	3	12	
CH240828001-2	3	3	3	3	12	
CH240828001-3	3	3	3	3	12	

**Table 4:** Comparison of the compliance rates between the gene chip and qPCR methods.

Virus	qPCR	Gene Chip	Compliance Rate
GPV	3/210	3/210	100%
DEV	7/210	10/210	98.6%
MDPV	15/210	17/210	98.6%
DHAV-1	20/210	24/210	98.1%
DHAV-3	14/210	14/210	100%
DTMUV	6/210	8/210	100%
NDRV	13/210	15/210	99%
DHAV-3 + NDRV	4/210	4/210	100%
DHAV-1 + DHAV-3	8/210	9/210	99.5%
MDPV + NDRV	3/210	3/210	100%
DTMUV + NDRV	1/210	2/210	99.5%
DEV + MDPV + DHAV-3 + NDRV	1/210	2/210	99.5%

Compliance rate (%) = (total number of clinical specimens tested−number of different results)/total number of clinical specimens tested × 100%.

## Data Availability

The data presented in this study are available in this manuscript.
